# Ultra-High-Field MRI in the Diagnosis and Management of Gliomas: A Systematic Review

**DOI:** 10.3389/fneur.2022.857825

**Published:** 2022-04-05

**Authors:** Annabelle Shaffer, Susanna S. Kwok, Anant Naik, Aaron T. Anderson, Fan Lam, Tracey Wszalek, Paul M. Arnold, Wael Hassaneen

**Affiliations:** ^1^Carle Illinois College of Medicine, University of Illinois at Urbana-Champaign, Champaign, IL, United States; ^2^Beckman Institute for Advanced Science and Technology, University of Illinois at Urbana-Champaign, Champaign, IL, United States; ^3^Carle Illinois Advanced Imaging Center, University of Illinois and Carle Health, Urbana, IL, United States; ^4^Department of Bioengineering, University of Illinois at Urbana-Champaign, Champaign, IL, United States; ^5^Cancer Center at Illinois, University of Illinois at Urbana-Champaign, Champaign, IL, United States; ^6^Carle Department of Neurosurgery, Carle Foundation Hospital, Urbana, IL, United States

**Keywords:** ultra-high field imaging, ultra-high field MRI, 3-Tesla MRI, 7-Tesla MRI, brain tumors, gliomas, glioblastoma

## Abstract

**Importance:**

Gliomas, tumors of the central nervous system, are classically diagnosed through invasive surgical biopsy and subsequent histopathological study. Innovations in ultra-high field (UHF) imaging, namely 7-Tesla magnetic resonance imaging (7T MRI) are advancing preoperative tumor grading, visualization of intratumoral structures, and appreciation of small brain structures and lesions.

**Objective:**

Summarize current innovative uses of UHF imaging techniques in glioma diagnostics and treatment.

**Methods:**

A systematic review in accordance with PRISMA guidelines was performed utilizing PubMed. Case reports and series, observational clinical trials, and randomized clinical trials written in English were included. After removing unrelated studies and those with non-human subjects, only those related to 7T MRI were independently reviewed and summarized for data extraction. Some preclinical animal models are briefly described to demonstrate future usages of ultra-high-field imaging.

**Results:**

We reviewed 46 studies (43 human and 3 animal models) which reported clinical usages of UHF MRI in the diagnosis and management of gliomas. Current literature generally supports greater resolution imaging from 7T compared to 1.5T or 3T MRI, improving visualization of cerebral microbleeds and white and gray matter, and providing more precise localization for radiotherapy targeting. Additionally, studies found that diffusion or susceptibility-weighted imaging techniques applied to 7T MRI, may be used to predict tumor grade, reveal intratumoral structures such as neovasculature and microstructures like axons, and indicate isocitrate dehydrogenase 1 mutation status in preoperative imaging. Similarly, newer imaging techniques such as magnetic resonance spectroscopy and chemical exchange saturation transfer imaging can be performed on 7T MRI to predict tumor grading and treatment efficacy. Geometrical distortion, a known challenge of 7T MRI, was at a tolerable level in all included studies.

**Conclusion:**

UHF imaging has the potential to preoperatively and non-invasively grade gliomas, provide precise therapy target areas, and visualize lesions not seen on conventional MRI.

## Introduction

Gliomas are tumors arising from glial cells—astrocytes, oligodendrocytes, microglia—and make up approximately one-third of primary central nervous system (CNS) tumors. Physiologically, glial cells contribute to many essential tasks, including supporting neuronal metabolic function, contributing to the blood-brain barrier, and myelinating axons (1). Gliomas are characterized by the glial cell type underlying the neoplastic change and biomarkers, such as key genes or altered molecular pathways (2). Additionally, like other tumors, gliomas are further graded *via* the World Health Organization (WHO) criteria that utilizes histological and molecular genotypic features to assess the degree of malignancy on a scale of CNS WHO grade 1 (low grade, slow growing) to 4 (high grade, very aggressive) (2, 3). However, many studies referenced here utilize terminology from prior WHO classification criteria.

Early and accurate identification of gliomas is critical in diagnostics, prognostics, and strategic tailoring of individual therapy. Therapy can include a combination of surgery, radiotherapy, and chemotherapy. This characterization is especially important to the treatment of highly aggressive tumors, such as glioblastoma (GBM), which has a <5% 5-year survival probability (4). Presently, tissue biopsy is required for tumor grading and genotyping whereby assessment of glial fibrillary acidic protein production, isocitrate dehydrogenase 1 (IDH) mutational status, presence of 1p/19q codeletion, epidermal growth factor receptor (EGFR) expression, E3 ubiquitin-protein-ligase-Mdm2 encoding gene mutation status, and other neoplastic changes within gliomas enables the more robust characterization that is critical to the development of individualized treatment associated with improved clinical outcomes (2–4). For example, IDH mutations have improved survival outcomes compared to IDH-wildtype (WT) (5), and O(6)-methylguanine-DNA methyltransferase (MGMT) promoter methylation is a favorable prognostic factor in GBM (6).

However, histopathology necessitates surgical collection of brain tissue and must be preceded by extensive clinical workup to confirm the presence of cancerous tissue. An integral part of clinical workup is brain imaging, ideally MRI, which is used to confirm the presence of a lesion, its location, and to generate a plan for intraoperative navigation (7). Traditional low field MRI (1.5T or 3T, found in most clinical settings) has been shown to significantly improve the extent of glioma resection (8). Advances in low field MRI acquisition and processing, including innovative uses of diffusion-weighted imaging (DWI) and magnetic resonance spectroscopic imaging (MRS/I) algorithms, have enabled both more accurate delineation of tumors margins and pre-operative assessment of biomolecular characteristics associated with gliomas that was previously only feasible through histologic assessment of biopsied brain tissue. These imaging sequences, along with numerous other advancements (4), have demonstrated the ability of MRI to provide information for enhanced tumor characterization and improved predictions of treatment efficacy that traditionally relied on surgical biopsy.

**Figure 1:**
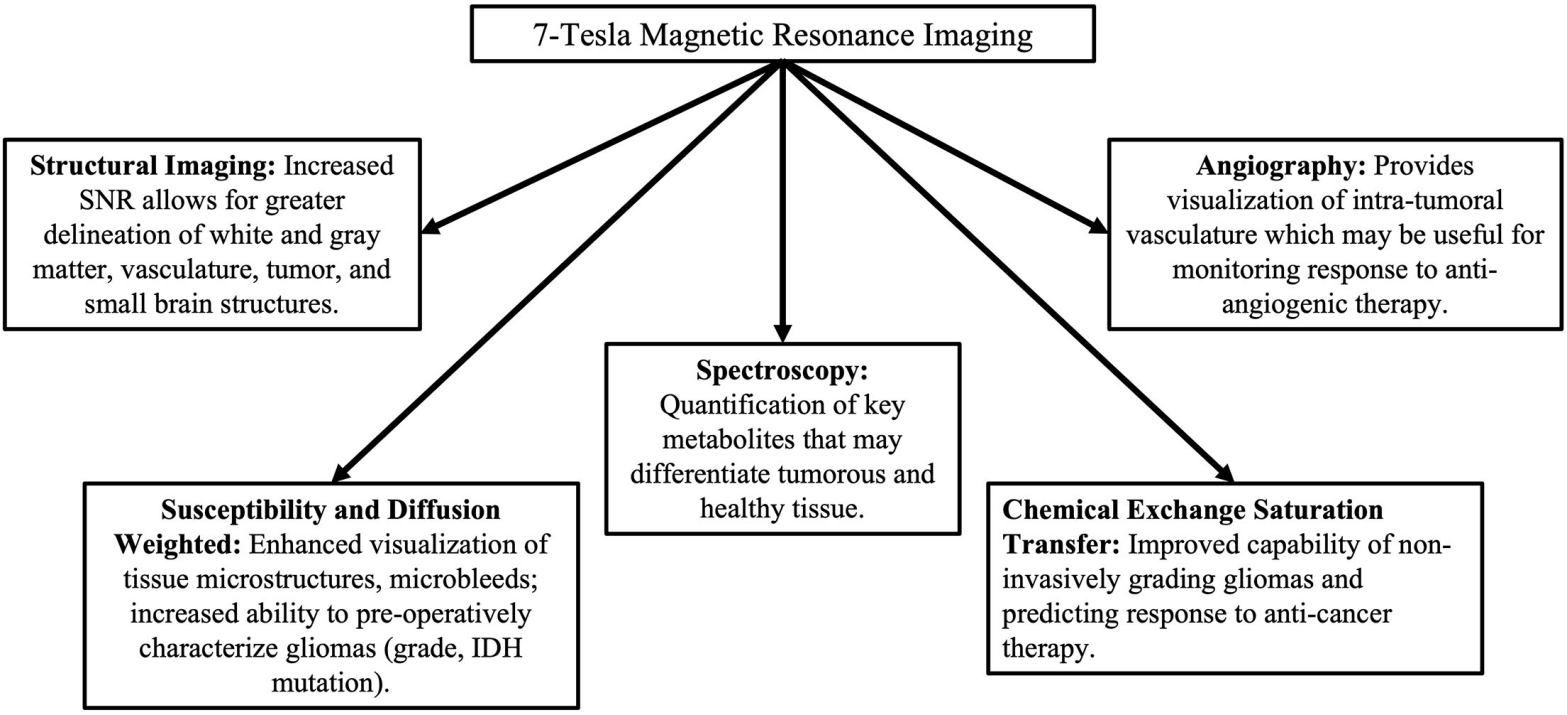
Types of advanced imaging techniques and their benefits in comparison to lower-field MRI.

Ultra-high field (UHF) MRI systems are defined as having a magnetic field strength of 7T or higher (9, 10) and different applications are displayed in Figure 1. The enhanced sensitivity and stronger gradient systems have allowed for the acquisition of higher resolution images of the brain with clinically acceptable acquisition times (11), as well as more accurate quantification of tissue biophysical parameters and disease states (12). Variations in image acquisition and processing enable unique characterization of gliomas at UHF may improve diagnosis and treatment efficacy prediction without the need for tissue biopsy. Numerous studies have demonstrated the utility of UHF imaging on patients with glioma. To further our understanding of the potential clinical benefits of UHF imaging on gliomas, we seek to summarize such studies in this concise, systematic review.

### Ultra-High Field MRI: A Cost Benefit Analysis

UHF systems offer a variety of benefits in assessing tissue. First, the signal-to-noise ratio (SNR) has been found to scale up to super-linearly with static magnetic field strength (B0) enabling higher spatial resolution and enhanced gray vs. white matter contrast (10, 13–15). Second, UHF imaging provides higher resolution molecular data that can be used in the characterization of non-structural neoplastic changes (such as metabolic activity, angiogenesis, and protein synthesis) in the brain which, at low-field, has been severely limited by poor resolution and long acquisition time (16–19). Third, spectral resolution is enhanced due to elevated SNR and separation of metabolite peaks allowing for more metabolites to be detected (20).

The cost of UHF systems is significantly greater than lower field systems as each Tesla costs ~$1 million (9). Other upfront costs include larger space to house the scanner, reinforced flooring to support the weight of the magnet, and more steel for magnetic shielding. In our institution's experience of acquiring a 7T MRI, we have encountered increased service costs due to less expertise available for 7T MRI technical support and increased time required to optimize imaging protocols compared to lower field systems. Costs for cooling the superconducting magnet are unchanged due to its zero-boil-off nature which eliminates the need for helium refills. Hospital revenue for MRI scans is also unchanged as there is only one rate available. However, as 7T imaging becomes more specific for different pathologies, the need for other tests may decline, reducing the overall costs incurred.

## Methods

**Figure 2:**
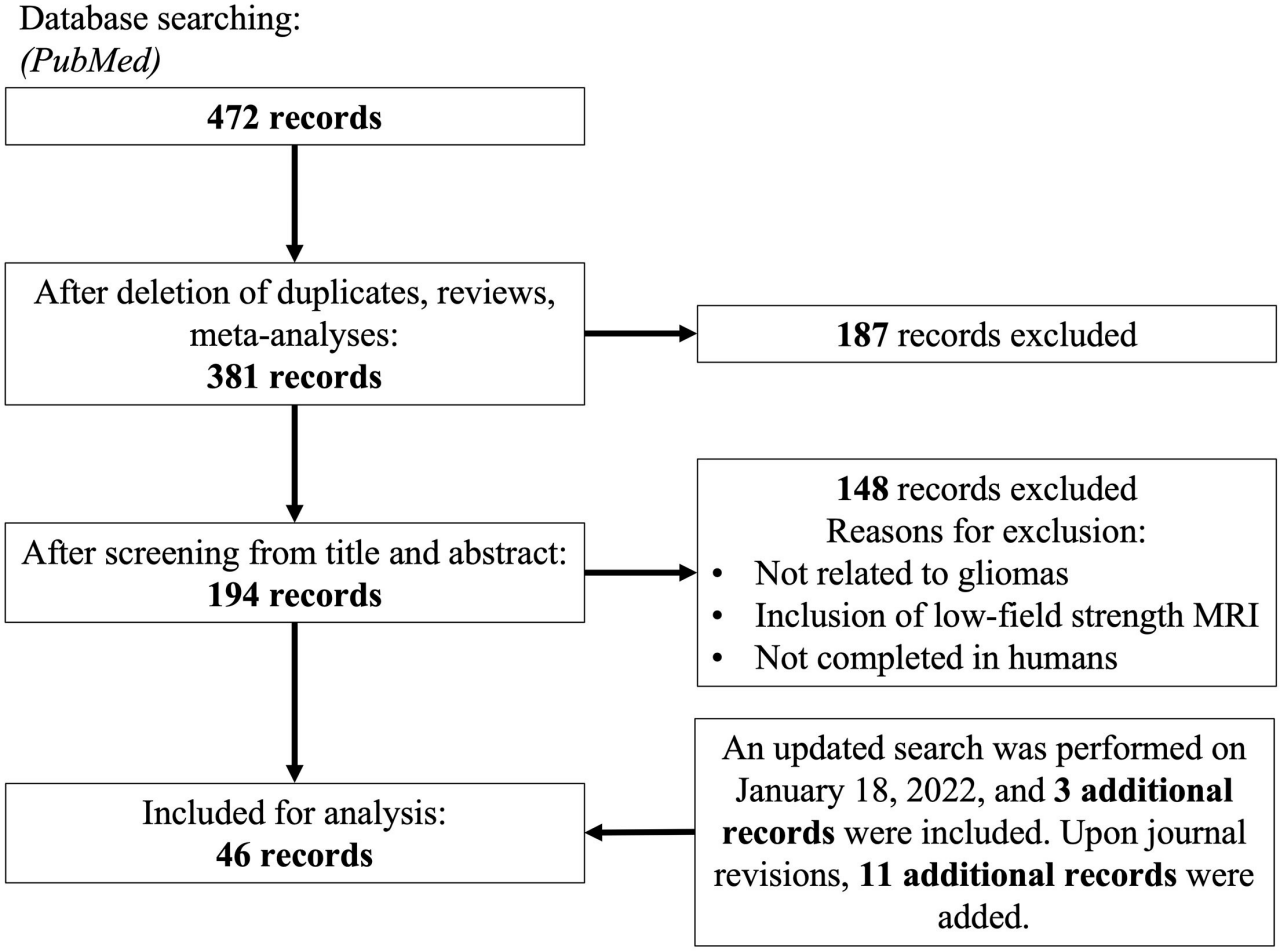
PRISMA diagram for search strategy.

We performed a systematic review adherent to PRISMA guidelines. A search on PubMED was performed on March 30th, 2021 using the following terms: *((Ultra high field) OR (“7-Tesla” OR “7Tesla” OR “7 Tesla”) OR (((“7T” OR “7-T” OR “7 T”) AND (MRI)))) AND ((Glioblastoma) OR (GBM) OR (Glioma))*. Articles were also subsequently added after citation matching and a more updated literature search. We sought to obtain articles that utilized various UHF MRI techniques for the characterization of glioma. We included case reports, case series, retrospective and prospective observational studies, and randomized controlled trials. Clinical evidence was prioritized over preclinical models. Non-full length and non-English records were excluded. No date constraints were imposed. Figure 2 summarizes the search strategy and subsequent inclusion and exclusion parameters utilized in this systematic review. Supplementary Table 1 summarizes characteristics of all included human studies. We examined several previously defined outcomes. First, we sought to investigate a comparable improvement in signal-to-noise ratio, yielding better diagnostic capacity across multiple imaging modalities. Second, we sought to expand diagnostic impact, which was defined by any possible alterations in clinical management by the use of UHF imaging. This included impact on patient mortality, progression free survival, degree of tumor resection, and selection of treatment modality post-operatively.

## Results

From an initial 472 articles obtained through databases, elimination of non-duplicates, reviews and meta-analyses yielded 381 articles whose title, abstract and additional meta-data were screened against the inclusion criteria defined. One hundred and ninety-four full-length articles were screened, yielding 46 for consideration for final meta-analysis, of which 11 articles were discovered with an updated search and citation matching.

### Structural Imaging

The advent of *in vivo* non-invasive structural imaging revolutionized medical practice, enabling the visualization of anatomic changes related to disease without autopsy. Due to its ability to provide high-contrast in soft tissue, MRI has been the cornerstone of assessing structural changes in the brain. Improvements to traditional, low-field structural imaging *via* UHF has improved SNR ratios, providing higher resolution contrast to enhance delineation of smaller white matter, gray matter, vasculature, and other brain structures (11, 21). Image acquisition at 7T can be clinically feasible with durations of 7:30 min and minimal side effects (11). Compter et al. (21) demonstrated the ability of 7T MRI to depict micro-vascularization in GBM. Furthermore, this study showed that incorporation of these images into neurosurgical navigation and radiotherapy treatment planning systems was not only technically feasible, but suggested that its use, following clinical-use optimization, may improve surgical outcomes compared to systems guided by low-resolution computed tomography images (21). In a study that assessed pre-operative GBM, Regnery et al. (11) compared fluid-attenuated inversion recovery (FLAIR) sequenced images obtained at 3T compared to 7T. This study found higher SNR in white matter and greater contrast of healthy white matter to GBM tissue with the subsequent conclusion that UHF FLAIR may enhance visualization of tumor infiltration into neighboring white matter tracts (11). They found demarcation of tumor boundaries through UHF significantly reduced calculated GBM volume by 7.4%, implying that UHF has the potential to spare more healthy brain tissue (11).

**Figure 3:**
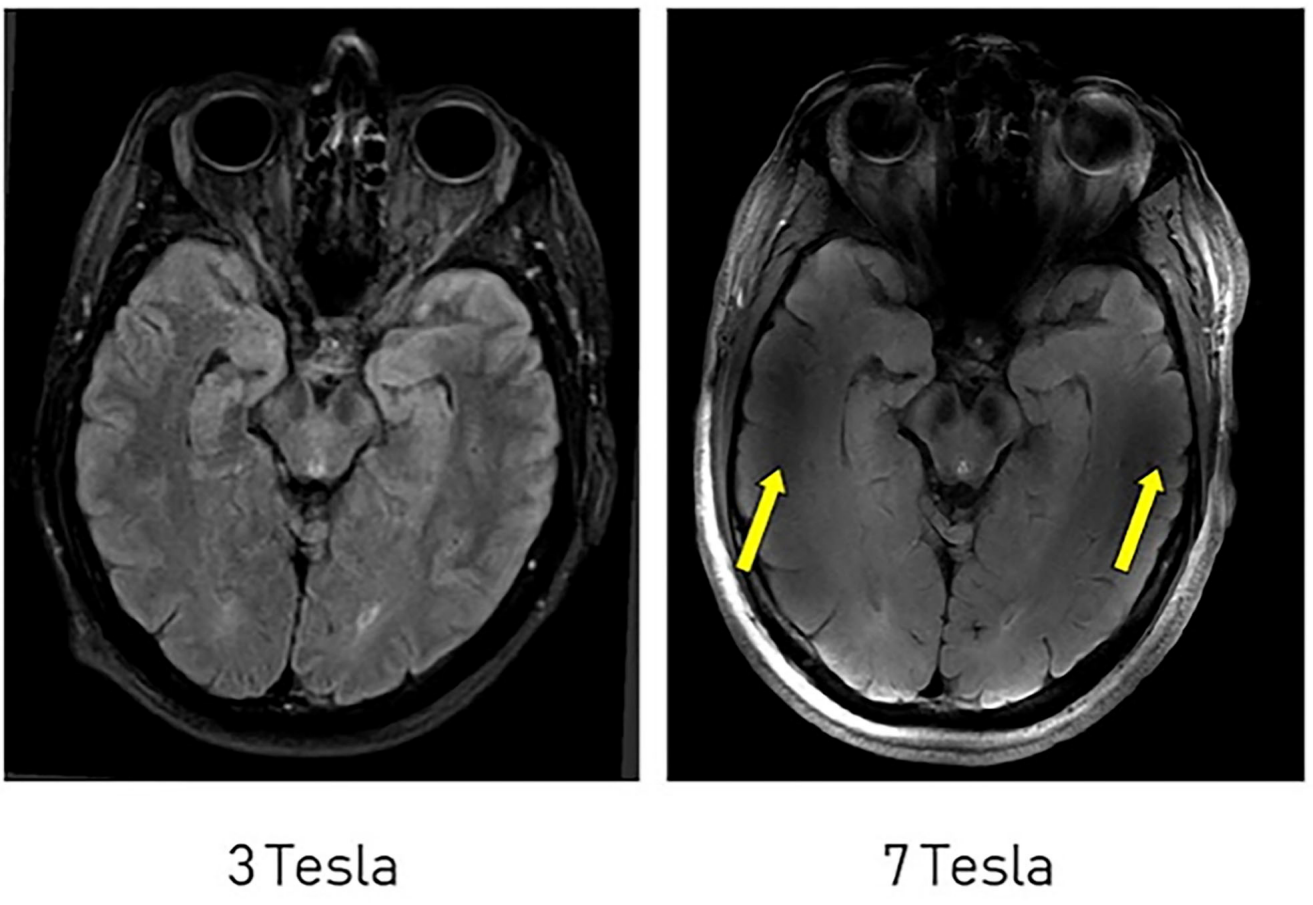
Comparison of FLAIR imaging at 3T and 7T. Artifacts are more significant near the skull base at 7T (yellow arrows). This figure is used with permission from Regnery et al. (11).

Challenges to structural imaging at 7T include poor image quality and the presence of ghosting artifacts in structures nearest the skull base (Figure 3), namely the frontal and temporal lobes, which may be attributed to signal heterogeneity caused by the transition of tissue type (21). These challenges, which are similarly described at lower field strengths, may be overcome through image correction specific to sequences obtained, usage of dielectric pads, and improvements in hardware (11, 21–23).

### Diffusion and Susceptibility-Weighted Imaging

Susceptibility weighted imaging (SWI) combines both the magnitude and phase components of MRI data to generate images, while standard diagnostic images only make use of magnitude information. SWI is sensitive to diamagnetic, paramagnetic, and ferromagnetic substances due to the phase shifts that these substances cause. DWI also uses MRI to visualize tissue microstructures, such as axons and glial cells, through the random motion of water particles. Both imaging techniques have been utilized to analyze small structures in the brain, such as microbleeds after radiation therapy and intra-tumoral vasculature. As outlined below, usage of these techniques could provide enhanced estimation of pre-operative tumor grade, characterization of tumor microstructures, and visualization of radiation therapy injury.

Currently, diagnosis of a glioma requires histopathological analysis of a surgical biopsy obtained. MRIs can also provide an estimation of a tumor's grade based on the contrast-enhancement present (24). However, MRI estimation of tumor grade is limited by inconsistency of contrast enhancement in benign and malignant gliomas and contraindications to the use of contrast (24). As such, new preoperative imaging techniques are being developed to estimate tumor grade more accurately.

Neovascularization is a distinguishing feature of highly malignant gliomas (25), such as GBM, and can be visualized through SWI. Venous structures can also be visualized *via* SWI 7T (26). Two case series (25, 27) utilized longitudinal SWI 7T MRI to identify changes in tumor vasculature during administration of an anti-angiogenic therapy, bevacizumab, to patients with a GBM or anaplastic astrocytoma. Grabner et al. (27) visualized changes in brain edema in four of five patients, microhemorrhages in one patient, and increased intratumoral irregularities in one patient. In another patient, intratumoral changes were difficult to visualize due to scarring (27). Similarly, SWI 7T MRI and fractal dimension analysis was used to quantify changes in intratumoral vasculature and complexity (25, 28). Fractal dimensions describe an object's geometric complexity and have been used in neuroradiology to detect lesions (25, 28). In a four patient case series, one patient had neurological improvements alongside a decreased fractal dimensions value following anti-angiogenic therapy (25). In a 36 patient series, a significant difference in fractal dimension values was identified between WHO grade 2 and grade 4 gliomas (28).

In addition to tumor vascularity, molecular markers, such as IDH, are useful in tumor grading and prognosis. IDH-mutant is associated with gliomas of lesser grades and greater progression-free survival time (24). Median survival time of GBM patients with an IDH mutation was 16 months more than those with IDH-WT (15 vs. 31 months) (29). Grabner et al. (24) utilized local image variance (LIV) of hypo-intensities seen on SWI 7T MRI and compared LIV values to tumor grade and IDH-mutant status. In a 30 patient study, where half had an IDH mutation, LIV values were significantly increased in WHO grade 3 and 4 gliomas and significantly correlated with numerical grading. Furthermore, significantly higher LIV values were found in IDH-WT gliomas vs. IDH-mutant gliomas (24).

SWI MRI has also been utilized to assess radiation therapy-induced injury to the brain. Lupo et al. (30) and Bian et al. (31) assessed cerebral microbleeds after radiation therapy using 7T SWI MRI and found microbleeds only in glioma patients who had undergone radiation therapy. Glioma patients who had exclusive chemotherapy did not present with these lesions. Additionally, Bian et al. (31) compared microbleed detection in SWI 3T and SWI 7T MRI. SWI MRI at 3T and 7T strengths detected significantly more microbleeds than magnitude-only images. However, there were insignificant differences in microbleeds detected by SWI 7T MRI vs. SWI 3T MRI when both deep-brain (*n* = 3) and non-deep-brain tumors (*n* = 7) were considered (31). When only non-deep-brain tumors were considered, SWI 7T MRI detected significantly more microbleeds than SWI 3T MRI (31).

Due to DWI's ability to visualize tissue microstructures, it has been used to evaluate many brain disease processes including gliomas. Wen et al. (32) described the clinical feasibility of acquiring DWI 7T MRI and the comparability to images acquired with DWI at 3T MRI. The images at 7T provided unique contrast that supported better visualization of tissue complexity.

### Spectroscopy

Structural MRI uses signals from water protons (^1^H) to generate an anatomic depiction of the brain by voxel. It is also used to map data acquired through other modalities such as magnetic resonance spectroscopy (MRS). MRS has been traditionally used to identify biomolecules through ^1^H signals acquired in waveforms with unique resonance frequency differences. When MRS for a specified nucleus is collected voxel-by-voxel and mapped onto a structural MRI image, spectroscopy enables visualization of specific biomolecules within the brain as MRS/I. This enables the quantification and mapping of various molecules relevant to brain metabolism, possibly without exogenous contrast agents. Besides ^1^H, other nuclei can also be imaged by MRS/I, e.g., carbon (^13^C), deuterium (^2^H), fluorine (^19^F), phosphorus (^31^P), and sodium (^23^Na) (latter two have FDA-approved coils) enabling broad biomolecular profiling or depiction of molecular concentrations within a volume both at a given timepoint and longitudinally. MRS/I generates output in the form of spatially-resolved MR spectra, from which the detection of specific molecules of interest and quantification of molecular composition can be performed. The use of non-^1^H nuclei, due to low *in-vivo* concentrations, has had limited success at lower field-strength MRI; however, the use of UHF offers the unique advantage of capturing small concentrations of non-^1^H nuclei at high resolution.

Cancer is associated with a myriad of biomolecular changes that, in theory, make MRS/I uniquely suitable to non-invasively characterize tumors and contribute to treatment planning (33). However, the clinical applications of MRS/I have been greatly hindered by long acquisition times, low image resolution, and poor SNR. The improved instrumentation offered by UHF systems provides unique opportunities to address these long-standing challenges. Combined with the results from studies optimizing image acquisition and processing, 7T MRS/I has the potential to provide robust biomolecular information that could improve clinical diagnostics, evaluation of progression, and assessment of therapeutic responses of gliomas.

Currently, studies on 7T MRS/I in brain tumors can be categorized as either those that evaluate the efficacy of non-^1^H-MRS in tumor characterization (34–38) or those that modify image acquisition or processing methods to optimize ^1^H-MRS for clinical use (namely to increase SNR and decrease acquisition time) (39–43). Apart from ^1^H, 7T MRS/I using ^17^O, ^31^P, ^35^Cl, and ^23^Na NMR have been acquired on patients with gliomas to evaluate feasibility compared to existing methods (34–37). The use of non-^1^H nuclei enables the study of specific pathophysiologic processes involved in neurologic cancers: ^17^O allows for the depiction of oxygen-consumption correlated to changes in cerebral metabolic rate associated with glioma (44, 45); ^31^P enables visualization of ATP^1^ and pH (46, 47); ^35^Cl and ^23^Na enable the visualization of ion-pump and ion-channel activity related to electrochemical conduction (35, 36). Additionally, ^23^Na has demonstrated the ability to reflect both IDH mutation status and tumor grade (48). However, another study showed that ^23^Na in 7T MRI could not predict progression-free nor overall survival, but changes in total sodium content in GBM patients undergoing chemoradiotherapy were appreciated (49). Additional studies have sought to identify biomolecules specifically associated with gliomas using metabolic mapping on 7T MRS/I compared to 3T MRS/I (38, 39). These studies found that metabolites, including creatinine, choline, glutamate, glutamine, glutathione, myoinositol, N-acetylaspartate (NAA), N-acetyl-aspartylglutamine (NAAG), and taurine, could be visualized with 7T MRS/I (38, 39). Differences in metabolic profiles may distinguish tumorous tissue and healthy brain tissue (50).

UHF ^1^H-MRS/I studies also focus on overcoming the limitations of 3T MRS/I through adopting better instrumentation and tailored acquisitions such as the use of different MRI head coils (36), use of short and ultrashort echo times (40–43, 51), better localization (42), dual-readout alternated gradients (51), and adiabatic selective refocusing (43). Several of these studies utilize ^1^H-MRS/I to specifically evaluate 2-hydroxyglutarate (2HG) due to its up-regulation in patients with an IDH mutation (38, 42, 43, 51). GBMs with IDH-mutant status have increased NADPH activity which increases the production of 2HG and may subsequently be associated with more favorable outcomes (38, 42, 43, 51, 52). Overall, these studies show that enhanced visualization of 2HG can be obtained. Optimization of ^1^H-MRS/I allows for detection of 2HG in concentrations as low as 0.5 mM (52). Therefore, further optimizations and improvements may achieve higher resolution and better robustness to enable clinical use in diagnostics and prognostics.

Although the use of 7T MRS/I to differentiate between glioma recurrence vs. radiation necrosis has not been described in any currently published study, the use of MRS/I in 3T MRI can distinguish between glioma recurrence and radiation necrosis. For example, choline to NAA ratio, choline to creatine ratio, and choline to lipid ratio have been shown to be significantly elevated in glioma recurrence compared to radiation necrosis on 3T MRS in multiple studies (53, 54). Therefore, given the propensity of studies to have similar results when moved from 3T to 7T imaging, it is probable that UHF imaging will yield similar results with greater sensitivity and specificity. However, the exact results and the advantages that UHF MRS/I may provide will require future investigation. Additionally, given the difference in physiologic function between necrotic tissue and neoplastic tissue, MRS/I with other nuclei to assess ion channel function and biochemicals specific to cell growth, proliferation, and angiogenesis as opposed to those that are affiliated with cell death and apoptosis, may enhance the ability of MRS/I to delineate between glioma recurrence and radiation necrosis.

### Chemical Exchange Saturation Transfer

Chemical exchange saturation transfer (CEST) is a contrast enhancement technique that enables the detection of specific molecules containing amides, amines, or hydroxyl protons exchangeable with bulk water (19, 55–61). Depending on the molecule targeted for saturation and their *in-vivo* concentrations, CEST has multiple variants that are useful in tumor grading and prediction of treatment efficacy: amide-proton-transfer CEST (APT-CEST) that detects and images amide and amine groups, glutamate-CEST that images glutamate, D-glucose CEST (glucose-CEST) that images glucose, and acidoCEST that images pH changes in GBM. As CEST requires the application of frequency-selective radiofrequency (RF) radiation to the exchangeable proton groups, the increased frequency dispersion at UHF is expected to improve signal detection specificity. The molecular imaging nature of CEST shows promise for improved capability for tumor grading and treatment efficacy prediction. The asymmetrical MTR parameter is most commonly used for CEST quantification; however, it is affected by other CEST pools, such as nuclear Overhauser effects (NOE), and technical issues, such as direct water saturation and magnetization transfer contrast. More sophisticated quantification methods discussed here seek to accurately depict proton concentrations while maximizing sensitivity to microenvironmental changes (such as pH and temperature) *in-vivo* through the use of various fitting algorithms and corrections (62–64).

NOE mediated CEST at 7T MRI was compared to standard contrast-enhanced MR images in GBM patients (55). Compared to normal-appearing contralateral tissue, regions of tumor tissue, radiation necrosis, and peritumoral hyperintensities had significantly greater NOE-CEST signal (55). Additional areas of hyperintensity, not seen on standard imaging, were also identified using NOE-CEST, indicating NOE-CEST likely provides further structural detail (55). Similarly, Zaiss et al. (19) found that by utilizing a correction algorithm for apparent exchange-dependent relaxation (AREX), NOE-CEST shows a significantly greater contrast between tumorous and necrotic tissue and between tumor and healthy tissue. Utilization of amide-CEST in the same study did not result in significant contrast (19). Later, a follow-up study concluded that APT-CEST could generate unique contrast that overlaps with gadolinium weighted T1 imaging if it was corrected for AREX and downfield-aliphatic-related NOE signal was suppressed (56). However, NOE and other magnetization transfer effects have been shown to be significantly different in tumors compared to healthy tissue. These findings suggest that further elucidation of CEST differences and implementation of corrections are necessary to accurately visualize characteristic differences between gliomas and healthy tissue (56, 64).

CEST at 7T MRI has also been investigated for its ability to predict tumor grade, IDH mutation status, and MGMT promoter methylation. Comparing pre-surgical NOE- and APT-CEST 7T MR images and histopathological biopsy in 10 patients, Heo et al. (58) identified significantly less NOE-CEST signal in gliomas vs. contralateral healthy tissue (*p* < 0.01). Additionally, the NOE-CEST signal differed significantly in WHO grade 2 vs. WHO grade 3-4 gliomas (*p* < 0.05) (58). Differences in APT-CEST signals across tissue types were not appreciated (58). A similar study that analyzed biopsy samples from 15 patients with glioblastoma showed improved visualization of cellularity using NOE-CEST compared to diffusion-weighted MRI (65).

Utilizing downfield NOE-suppressed APT-CEST (dns-APT), Paech et al. (57) predicted IDH mutation status and differentiated gliomas of low and high grades but could not predict MGMT promoter methylation (57). APT-CEST signals were significantly greater in gliomas localized to the right hemisphere than the left (*p* < 0.05), and NOE-CEST signals were significantly greater in gliomas with subventricular zone contact (*p* < 0.05) (59). Healthy controls did not exhibit hemisphere differences in signals (59). While subventricular zone contact can indicate a poor prognosis, no histopathological characteristics, including cell density and ki67, were significantly associated with location (59). An inverse relationship between survival and APT values was demonstrated using relaxation-compensated APT-CEST (66). Furthermore, APT-CEST signal differences correlate with treatment response. Using pre-treatment, post-treatment, and 6 months post-treatment groups, Meissner et al. showed that 7T dns-APT CEST signal was significantly different between chemoradiotherapy treatment-responsive and treatment-non-responsive groups; however, this was only appreciable between the pre-treatment group and immediate post-treatment group (61).

**Figure 4:**
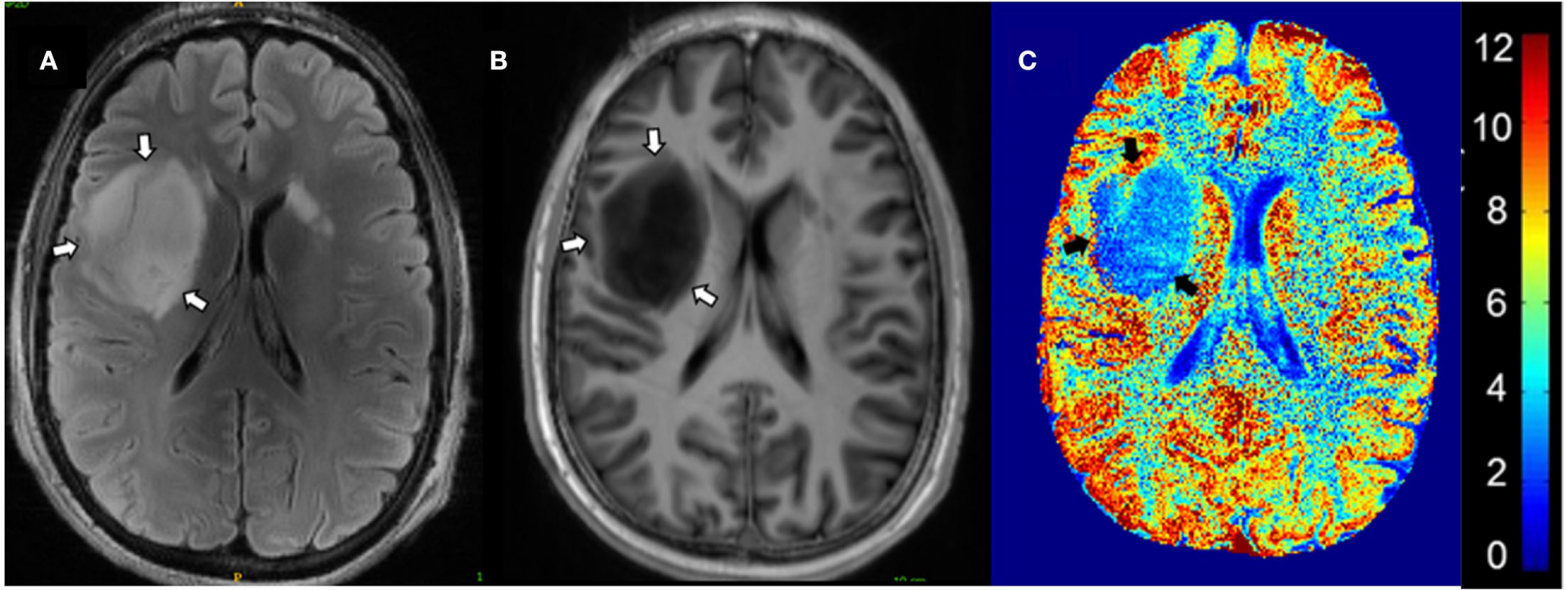
Glutamate-CEST imaging of a diffuse astrocytoma. **(A)** 3T FLAIR with tumors identified by white arrows. **(B)** 7T FLAIR with tumors identified by white arrows. **(C)** Glutamate-CEST contrast map with tumors identified by black arrows and CEST contrast gradient on the right. Figure used with permission from Neal et al., 2019 (60).

CEST 7T MRI may be a mechanism to predict clinical characteristics and anti-cancer treatment response. Neal et al. (60) conducted a study on the effectiveness of glutamate-CEST (Figure 4) to detect both the aggressiveness of the glioma and determine the risk of having glioma-associated seizures. Increased peritumoral glutamate-CEST contrast was significantly associated with recent seizure (*p* = 0.038) and drug-refractory epilepsy (*p* = 0.029) (60). This data suggests that glutamate-CEST contrast may indicate the likelihood of glioma-associated seizures.

Gadolinium-based contrast agents are frequently used in MRI of the brain and other tissues; however, there is renewed concern about its safety profile due to reports of nephrogenic systemic fibrosis and deposition of gadolinium in tissues. As such, there is interest in developing non-metallic and biodegradable contrast agents. D-glucose may offer a biodegradable contrast agent. Xu et al. (67) and Schuenke et al. (68) demonstrated the feasibility of gluco-CEST and glucose-CESL (chemical exchange sensitive spin-lock), respectively, at 7T in patients with gliomas. Paech et al. (69) found that glucose-CESL signals were significantly increased in tumor tissue compared to contralateral normal tissue and that areas of glucose-CESL intensity correlated to areas of tumor tissue seen on gadolinium-enhanced images (69). Case studies have also demonstrated better identification of tumor borders (68) and enhanced contrast between tumor and normal tissue (70) using glucose-CESL methods. Animal models of glucose-based agents are discussed in Preclinical Models and Future Directions.

### Angiography

There exists few studies utilizing angiography at 7T MRI to visualize tumoral vasculature in humans. Limited data exists in rodent models as well (71). Radbruch et al. (72) utilized time-of-flight angiography at 7T MRI to visualize tumor vessels in 12 patients with GBM. All patients were imaged prior to surgical resection or treatment with anti-cancer therapies. Tumor vessels were found to be clearly visible in all patients. Using multiscale vessel enhancement filtering and segmentation, tumor vasculature was characterized and compared to healthy white matter. Tumor vasculature had significantly greater vessel surface area (*p* < 0.035) and number of branches (*p* < 0.001) but had lesser vessel length (*p* < 0.001). It was also observed that tumors had an insignificantly greater density of vessels (*p* < 0.078). Visualization and quantification of tumor vasculature could offer a monitoring method for anti-angiogenic therapy and tumor grading.

### Preclinical Models and Future Directions

Murine model studies are currently underway to improve imaging and non-invasive tumor characterization. Sehgal et al. (73) investigated the use of 3-O-methyl-D-glucose (3-OMG), a non-metabolizable analog of glucose, as a contrast agent in MRI studies using CEST. In a murine glioma model, contrast enhancement using 3-OMG was found to be nearly double the contrast enhancement seen when using D-glucose (2.5–5.0% vs. 1.5–3.0%). Unlike gadolinium agents that require a disrupted blood brain barrier to diffuse into the brain, glucose transporters in the brain can uptake 3-OMG. Being non-metabolized, 3-OMG is not affected by metabolism or insulin's response. While a toxicity profile of infused 3-OMG is needed, it has been used orally in human nutrient absorption studies indicating it is likely without evidence of safety issues.

In a murine study utilizing 11.7T MRI to study a variety of tumors (glioma, neuroblastoma, and breast cancer cell lines), Tanoue et al. (74) found differences in APT signaling based on tumor type and histopathological characteristics. It was found that APT signaling was elevated in tumors with greater proliferative potential. Additionally, regions of bleeding also correlated to regions of elevated APT signaling.

A critical challenge of GBM is recurrence due to its infiltrative nature. Cellular infiltration into healthy regions is difficult to quantify using conventional MRI. Utilizing 7T MRI perfusion images, Vallatos et al. (75) demonstrated a negative correlation between tumor cell infiltration and perfusion near tumor margins. Perfusion imaging identified a larger abnormal brain region surrounding the murine GBM compared to relaxation and diffusion-based techniques. Perfusion imaging was more sensitive to abnormal tissue, but less specific than other modalities. Perfusion imaging techniques may provide a therapy target region that is more inclusive of remote cellular infiltrate although further work is necessary.

## Limitations

In addition to added costs and limited availability, several limitations exist for 7T MRI use in gliomas. 7T MRI can contribute to patient side effects, such as peripheral nerve stimulation, localized heating, vestibular effects, and increased RF energy deposition in tissues which carries unknown long term health effects (21, 76). Poor image quality and image distortions are often seen, especially when imaging structures deep within the brain and nearest the skull base (21, 31). These distortions are related to differences of magnetic susceptibility between air cavities of the skull and brain (77). Motion artifacts are frequently seen because the increased SNR in 7T MRI also increases its sensitivity to patient motion during the scan, such as breathing and heartbeats (77). SNR decreases from the center to the periphery of the brain (77). RF head coils, such as the commercial distributed 1-channel quadrature birdcage 32-channel receive head coil by Nova Medical (Wilmington, MA) and the pTx version for research purposes, allow for increased SNR (78). Innovations in head coil designs are also underway to better visualize cerebellum structures (78). Lastly, advanced imaging analysis, such as fractal dimensions, is likely infeasible in current clinical settings.

## Data Availability Statement

The original contributions presented in the study are included in the article/Supplementary Material, further inquiries can be directed to the corresponding authors.

## Author Contributions

AS, SK, and AN contributed to conception of the study and execution of systematic review. AA, FL, TW, PA, and WH contributed to critical revisions of the manuscript. All authors contributed to the article and approved the submitted version.

## Conflict of Interest

The authors declare that the research was conducted in the absence of any commercial or financial relationships that could be construed as a potential conflict of interest.

## Publisher's Note

All claims expressed in this article are solely those of the authors and do not necessarily represent those of their affiliated organizations, or those of the publisher, the editors and the reviewers. Any product that may be evaluated in this article, or claim that may be made by its manufacturer, is not guaranteed or endorsed by the publisher.
