# Supplementary material for: Ultra-High-Field MRI in the Diagnosis and Management of Gliomas: A Systematic Review
Source: Front Neurol. 2022 Apr 5;13:857825. doi: 10.3389/fneur.2022.857825 (PMC9016277; doi:10.3389/fneur.2022.857825)
Supplement: Supplementary file 1 [file Table_1.DOCX]

| **Study** | **Year** | **Imaging modality; 7T manufacturer** | **Sequences utilized (7T)** | **Patients (n)** | **Disease(s) included** | **Scan frequency** |
| --- | --- | --- | --- | --- | --- | --- |
| Compter et al(1) | 2016 | Structural; Siemens Healthcare (Magnetom 7T) | MP2RAGE, T2-SPACE, SPACE-FLAIR, GRE | 3 | Healthy volunteers^ | 1 scan |
| Regnery et al(2) | 2019 | Structural; Siemens Healthcare (Magnetom 7T) | FLAIR | 15 | Glioblastoma | 2 scans*: 1 3T and 1 7T |
| Grabner et al(3) | 2012 | Susceptibility weighted imaging (SWI); not reported (N/R) | MPRAGE, 3D GRE | 5 | Glioblastoma and anaplastic astrocytoma treated with bevacizumab | 3 scans: baseline, 2 weeks, 4 weeks |
| Di Ieva et al(4) | 2012 | SWI; Siemens Healthcare (Magnetom 7T) | 3D GRE | 4 | Glioblastoma and anaplastic astrocytoma treated with bevacizumab | 3 scans: baseline, 2 weeks, 4 weeks |
| Di Ieva et al(5) | 2013 | SWI; Siemens Healthcare (Magnetom 7T) | MPRAGE, 3D GRE | 36 | Astrocytoma, oligodendroglioma, oligoastrocytoma, glioblastoma | 1 scan |
| Grabner et al(6) | 2017 | SWI; Siemens Healthcare (Magnetom 7T) | MPRAGE | 30 | Glioblastoma, gliosarcoma, anaplastic astrocytoma, anaplastic oligodendroglioma, mixed oligoastrocytoma, oligodendroglioma, diffuse astrocytoma | 1 scan |
| Bian et al(7) | 2013 | SWI; GE Healthcare Technologies | 3D GRE | 10 | Patients with previous glioma who had radiation therapy | 2 scans: 1 3T and 1 7T |
| Lupo et al(8) | 2012 | SWI; GE Healthcare Technologies | 3D SPGR, 2D GRE | 25 | Astrocytoma, oligodendroglioma, mixed oligoastrocytoma, ependymoma, anaplastic astrocytoma, anaplastic oligodendroglioma, mixed anaplastic oligoastrocytoma, glioblastoma | 2 scans: 1 3T and 1 7T |
| Wen et al(9) | 2015 | DWI; GE Healthcare Technologies (MR950) | MB NODDI (neurite orientation dispersion and density imaging) | 25 | Healthy volunteers (n=5), glioma grades 2-4 (n=20) | 2 scans: 1 3T and 1 7T |
| Gruber et al(10) | 2017 | High resolution free induction decay MRS; Siemens Healthcare (Magnetom 7T) | MP2RAGE,  turbo-FLASH–based B_1_^+^-mapping | 12 | Anaplastic oligoastrocytoma (n=1), oligodendroglioma (n=1), healthy volunteers (n=10) | 2 scans: 1 3T and 1 7T |
| Hangel et al(11) | 2019 | High resolution free induction decay MRS; Siemens Healthcare (Magnetom 7T) | MPRAGE, MP2RAGE, FLAIR, SWI, B_1_^+^-mapping | 10 | Oligodendroglioma, anaplastic oligodendroglioma, diffuse astrocytoma, glioblastoma | 2 scans: 1 3T and 1 7T |
| Li et al(12) | 2015 | ^1^H-MRS; GE Healthcare Technologies (MR950) | 2D GRE, 2D FSE | 29 | Glioma grades 2-4 | 1 scan |
| Ganji et al(13) | 2017 | ^1^H-MRS; Philips Medical Systems (Achieva 7.0T) | FLAIR, PRESS | 12 | Oligoastrocytoma, oligodendroglioma, anaplastic astrocytoma, astrocytoma, anaplastic oligoastrocytoma | 1 scan |
| An et al(14) | 2018 | ^1^H-MRS; Philips Medical Systems (Achieva 7.0T) | FLAIR, PRESS | 5 | Oligodendroglioma, oligoastrocytoma, glioblastoma | 1 scan |
| Berrington et al(15) | 2018 | ^1^H-MRS; Siemens Healthcare (Magnetom 7T) | MPRAGE, semi-LASER | 9 | Astroglial tumor, glioblastoma, oligodendroglioma, anaplastic astrocytoma, diffuse astrocytoma | 2 scans: 1 3T and 1 7T |
| Esmaeili et al(16) | 2020 | ^1^H-MRS; Siemens Healthcare (Magnetom 7T) | FLAIR, MPRAGE, GRE, DESS, ASE | 4 | Healthy volunteers (n=3), IDH1-mutant glioma (n=1) | 1 scan |
| Shams et al(17) | 2021 | ^1^H-MRS; Philips Medical Systems (Achieva 7.0T) | sLASER, MEGA-sLASER, FLAIR | 4 | Diffuse astrocytoma, anaplastic astrocytoma | 1 scan |
| Bogner et al(18) | 2011 | ^31^P-MRS; Siemens Healthcare (Magnetom 7T) | ISIS (image selected *in vivo* spectroscopy) | 6 | Healthy volunteers | 2 scans: 1 3T and 1 7T |
| Ren et al(19) | 2015 | ^31^P-MRS; Philips Medical Systems (Achieva 7.0T) | EBIT | 12 | Healthy volunteers | 1 scan |
| Ren et al(20) | 2018 | ^31^P-MRS; Philips Medical Systems (Achieva 7.0T) | Non-localized pulse sequence | 11 | Healthy volunteers | 1 scan |
| Nagel et al(21) | 2014 | ^35^Cl, ^23^Na-MRS; Siemens Healthcare (Magnetom 7T) | Density-adapted 3D reconstruction double echo and IR, 3D GRE | 9 | Glioblastoma (n=2), healthy volunteers (n=7) | 1 scan |
| Biller et al(22) | 2016 | ^23^Na-MRS; Siemens Healthcare (Magnetom 7T) | Density-adapted 3D radial projection reconstruction pulse sequences | 34 | Oligodendroglioma, anaplastic oligodendroglioma, gliomatosis cerebri, anaplastic ependymoma, glioblastoma, metastasis | 2 scans: 1 3T and 1 7T |
| Regnery et al(23) | 2020 | ^23^Na-MRI; Siemens Healthcare (Magnetom 7T) | Density-adapted 3D radial pulse sequence | 28 | Oligodendroglioma, astrocytoma, glioblastoma | 2 scans: 1 3T and 1 7T |
| Paech et al(24) | 2021 | ^23^Na-MRI; Siemens Healthcare (Magnetom 7T) | Density-adapted 3D radial pulse sequence | 20 | Glioblastoma | 3 7T scans: baseline, immediately after chemoradiotherapy (CRT), 6 weeks after CRT; 3 3T scans: baseline, 4 weeks and 12 weeks post CRT |
| Hoffmann et al(25) | 2011 | ^17^O-MRI; Siemens Healthcare (Magnetom 7T) | 3D radial pulse | 1 | Healthy volunteer | 1 scan |
| Paech et al(26) | 2020 | ^17^O-MRI; Siemens Healthcare (Magnetom 7T) | Not reported | 13 | Glioblastoma (n=4), anaplastic oligodendroglioma (n=1), diffuse astrocytoma (n=5), healthy volunteers (n=3) | 1 scan |
| Meissner et al(27) | 2019 | CEST; Siemens Healthcare (Magnetom 7T) | GRE (gradient echo) | 12 | Glioblastoma, oligodendroglioma, astrocytoma | 4 scans: 1 3T and 1 7T at baseline, 7T at 1 week post CRT, and 7T 6 weeks post CRT |
| Zaiss et al(28) | 2015 | CEST; Siemens Healthcare (Magnetom 7T) | 2D GRE | 10 | Glioblastoma | 2 scans: 1 3T and 1 7T |
| Dreher et al(29) | 2019 | CEST; Siemens Healthcare (Magnetom 7T) | 2D GRE | 32 | Glioblastoma (n=20), gliosarcoma (n=1), healthy volunteers (n=11) | 2 scans: 1 3T and 1 7T |
| Paech et al(30) | 2018 | CEST; Siemens Healthcare (Magnetom 7T) | 2D GRE | 31 | Glioblastoma, gliosarcoma, anaplastic astrocytoma, oligodendroglioma, astrocytoma | 2 scans: 1 3T and 1 7T |
| Heo et al(31) | 2016 | CEST; Philips Medical Systems (Achieva 7.0T) | 3D GRE, MPRAGE | 10 | Oligodendroglioma, oligoastrocytoma, anaplastic astrocytoma, astrocytoma, glioblastoma | 1 scan |
| Windschuh et al(32) | 2015 | CEST; Siemens Healthcare (Magnetom 7T) | 2D GRE | 2 | Oligodendroglioma (n=1), healthy volunteer (n=1) | 1 scan |
| Zaiss et al(33) | 2017 | Amide-CEST; Siemens Healthcare (Magnetom 7T) | 2D GRE | 11 | Glioblastoma | 2 scans: 1 3T and 1 7T |
| Liu et al(34) | 2013 | Amide-, NOE-CEST; Siemens Healthcare (Magnetom 7T) | Not reported | 7 | Healthy volunteers | 1 scan |
| Paech et al(35) | 2014 | NOE-CEST; Siemens Healthcare (Magnetom 7T) | 3D GRE | 12 | Glioblastoma | 2 scans: 1 3T and 1 7T |
| Paech et al(36) | 2015 | NOE-CEST; Siemens Healthcare (Magnetom 7T) | 3D GRE | 15 | Glioblastoma | 2 scans: 1 3T and 1 7T |
| Paech et al(37) | 2019 | Amide-, NOE-CEST; Siemens Healthcare (Magnetom 7T) | 2D GRE | 26 | Glioblastoma, anaplastic astrocytoma | 2 scans: 1 3T and 1 7T |
| Neal et al(38) | 2019 | Glutamate CEST; Siemens Healthcare (Magnetom 7T) | MP2RAGE, 2D GRE | 10 | Astrocytoma, oligodendroglioma, anaplastic oligoastrocytoma | 2 scans^+^: 1 3T and 1 7T |
| Xu et al(39) | 2015 | Glucose-CEST; Philips Medical Systems (Achieva 7.0T) | GRE | 7 | Anaplastic astrocytoma (n=1), astrocytoma (n=1), glioblastoma (n=1), healthy volunteers (n=4) | 1 scan |
| Paech et al(40) | 2017 | Glucose-CESL; Siemens Healthcare (Magnetom 7T) | GRE, MPRAGE | 13 | Glioblastoma (n=9), healthy volunteers (n=4) | 1 scan |
| Schuenke et al(41) | 2017 | Glucose-CESL; Siemens Healthcare (Magnetom 7T) | 2D GRE | 2 | Gliosarcoma, glioblastoma | 1 scan |
| Schuenke et al(42) | 2017 | Glucose-CESL; Siemens Healthcare (Magnetom 7T) | TSE, GRE | 1 | Glioblastoma | 2 scans: 1 3T and 1 7T |
| Radbruch et al(43) | 2014 | Time of flight angiography; Siemens Healthcare (Magnetom 7T) | FLASH | 12 | Glioblastoma | 2 scans: 1 3T and 1 7T |

**Supplemental table 1.** 7T imaging studies performed in humans: 32 studies, 469 patients. In addition to the sequences listed, nearly all studies utilized T1 and T2 imaging, as such we declined to list these sequences.

^In all studies, healthy volunteers were defined as adults >18 years old and without any disease.

*Comparison shown in figure 3.

^+^Comparison shown in figure 4.

1. Compter I, Peerlings J, Eekers DB, Postma AA, Ivanov D, Wiggins CJ, et al. Technical feasibility of integrating 7 T anatomical MRI in image-guided radiotherapy of glioblastoma: a preparatory study. *MAGMA* (2016) 29(3):591-603. Epub 2016/03/31. doi: 10.1007/s10334-016-0534-7. PubMed PMID: 27026245.

2. Regnery S, Knowles BR, Paech D, Behl N, Meissner JE, Windisch P, et al. High-resolution FLAIR MRI at 7 Tesla for treatment planning in glioblastoma patients. *Radiother Oncol* (2019) 130:180-4. Epub 2018/09/05. doi: 10.1016/j.radonc.2018.08.002. PubMed PMID: 30177373.

3. Grabner G, Nobauer I, Elandt K, Kronnerwetter C, Woehrer A, Marosi C, et al. Longitudinal brain imaging of five malignant glioma patients treated with bevacizumab using susceptibility-weighted magnetic resonance imaging at 7 T. *Magn Reson Imaging* (2012) 30(1):139-47. Epub 2011/10/11. doi: 10.1016/j.mri.2011.08.004. PubMed PMID: 21982163.

4. Di Ieva A, Matula C, Grizzi F, Grabner G, Trattnig S, Tschabitscher M. Fractal analysis of the susceptibility weighted imaging patterns in malignant brain tumors during antiangiogenic treatment: technical report on four cases serially imaged by 7 T magnetic resonance during a period of four weeks. *World Neurosurg* (2012) 77(5-6):785 e11-21. Epub 2011/11/29. doi: 10.1016/j.wneu.2011.09.006. PubMed PMID: 22120276.

5. Di Ieva A, God S, Grabner G, Grizzi F, Sherif C, Matula C, et al. Three-dimensional susceptibility-weighted imaging at 7 T using fractal-based quantitative analysis to grade gliomas. *Neuroradiology* (2013) 55(1):35-40. Epub 2012/08/21. doi: 10.1007/s00234-012-1081-1. PubMed PMID: 22903580.

6. Grabner G, Kiesel B, Wohrer A, Millesi M, Wurzer A, God S, et al. Local image variance of 7 Tesla SWI is a new technique for preoperative characterization of diffusely infiltrating gliomas: correlation with tumour grade and IDH1 mutational status. *Eur Radiol* (2017) 27(4):1556-67. Epub 2016/06/15. doi: 10.1007/s00330-016-4451-y. PubMed PMID: 27300198; PubMed Central PMCID: PMCPMC5334387.

7. Bian W, Hess CP, Chang SM, Nelson SJ, Lupo JM. Susceptibility-weighted MR imaging of radiation therapy-induced cerebral microbleeds in patients with glioma: a comparison between 3T and 7T. *Neuroradiology* (2014) 56(2):91-6. Epub 2013/11/28. doi: 10.1007/s00234-013-1297-8. PubMed PMID: 24281386; PubMed Central PMCID: PMCPMC4940363.

8. Lupo JM, Chuang CF, Chang SM, Barani IJ, Jimenez B, Hess CP, et al. 7-Tesla susceptibility-weighted imaging to assess the effects of radiotherapy on normal-appearing brain in patients with glioma. *Int J Radiat Oncol Biol Phys* (2012) 82(3):e493-500. Epub 2011/10/18. doi: 10.1016/j.ijrobp.2011.05.046. PubMed PMID: 22000750; PubMed Central PMCID: PMCPMC3268881.

9. Wen Q, Kelley DA, Banerjee S, Lupo JM, Chang SM, Xu D, et al. Clinically feasible NODDI characterization of glioma using multiband EPI at 7 T. *Neuroimage Clin* (2015) 9:291-9. Epub 2015/10/29. doi: 10.1016/j.nicl.2015.08.017. PubMed PMID: 26509116; PubMed Central PMCID: PMCPMC4579286.

10. Gruber S, Heckova E, Strasser B, Povazan M, Hangel GJ, Minarikova L, et al. Mapping an Extended Neurochemical Profile at 3 and 7 T Using Accelerated High-Resolution Proton Magnetic Resonance Spectroscopic Imaging. *Invest Radiol* (2017) 52(10):631-9. Epub 2017/05/02. doi: 10.1097/RLI.0000000000000379. PubMed PMID: 28459799.

11. Hangel G, Jain S, Springer E, Heckova E, Strasser B, Povazan M, et al. High-resolution metabolic mapping of gliomas via patch-based super-resolution magnetic resonance spectroscopic imaging at 7T. *Neuroimage* (2019) 191:587-95. Epub 2019/02/18. doi: 10.1016/j.neuroimage.2019.02.023. PubMed PMID: 30772399; PubMed Central PMCID: PMCPMC7220803.

12. Li Y, Larson P, Chen AP, Lupo JM, Ozhinsky E, Kelley D, et al. Short-echo three-dimensional H-1 MR spectroscopic imaging of patients with glioma at 7 Tesla for characterization of differences in metabolite levels. *J Magn Reson Imaging* (2015) 41(5):1332-41. Epub 2014/06/18. doi: 10.1002/jmri.24672. PubMed PMID: 24935758; PubMed Central PMCID: PMCPMC4269580.

13. Ganji SK, An Z, Tiwari V, McNeil S, Pinho MC, Pan E, et al. In vivo detection of 2-hydroxyglutarate in brain tumors by optimized point-resolved spectroscopy (PRESS) at 7T. *Magn Reson Med* (2017) 77(3):936-44. Epub 2016/03/19. doi: 10.1002/mrm.26190. PubMed PMID: 26991680; PubMed Central PMCID: PMCPMC5026542.

14. An Z, Tiwari V, Ganji SK, Baxter J, Levy M, Pinho MC, et al. Echo-planar spectroscopic imaging with dual-readout alternated gradients (DRAG-EPSI) at 7 T: Application for 2-hydroxyglutarate imaging in glioma patients. *Magn Reson Med* (2018) 79(4):1851-61. Epub 2017/08/24. doi: 10.1002/mrm.26884. PubMed PMID: 28833542; PubMed Central PMCID: PMCPMC5811378.

15. Berrington A, Voets NL, Larkin SJ, de Pennington N, McCullagh J, Stacey R, et al. A comparison of 2-hydroxyglutarate detection at 3 and 7 T with long-TE semi-LASER. *NMR Biomed* (2018) 31(3). Epub 2018/01/10. doi: 10.1002/nbm.3886. PubMed PMID: 29315915.

16. Esmaeili M, Stockmann J, Strasser B, Arango N, Thapa B, Wang Z, et al. An integrated RF-receive/B0-shim array coil boosts performance of whole-brain MR spectroscopic imaging at 7 T. *Sci Rep* (2020) 10(1):15029. Epub 2020/09/16. doi: 10.1038/s41598-020-71623-5. PubMed PMID: 32929121; PubMed Central PMCID: PMCPMC7490394.

17. Shams Z, van der Kemp WJM, Emir U, Dankbaar JW, Snijders TJ, de Vos FYF, et al. Comparison of 2-Hydroxyglutarate Detection With sLASER and MEGA-sLASER at 7T. *Front Neurol* (2021) 12:718423. Epub 2021/09/25. doi: 10.3389/fneur.2021.718423. PubMed PMID: 34557149; PubMed Central PMCID: PMCPMC8452903.

18. Bogner W, Chmelik M, Andronesi OC, Sorensen AG, Trattnig S, Gruber S. In vivo 31P spectroscopy by fully adiabatic extended image selected in vivo spectroscopy: a comparison between 3 T and 7 T. *Magn Reson Med* (2011) 66(4):923-30. Epub 2011/03/30. doi: 10.1002/mrm.22897. PubMed PMID: 21446033.

19. Ren J, Sherry AD, Malloy CR. (31)P-MRS of healthy human brain: ATP synthesis, metabolite concentrations, pH, and T1 relaxation times. *NMR Biomed* (2015) 28(11):1455-62. Epub 2015/09/26. doi: 10.1002/nbm.3384. PubMed PMID: 26404723; PubMed Central PMCID: PMCPMC4772768.

20. Ren J, Shang T, Sherry AD, Malloy CR. Unveiling a hidden (31) P signal coresonating with extracellular inorganic phosphate by outer-volume-suppression and localized (31) P MRS in the human brain at 7T. *Magn Reson Med* (2018) 80(4):1289-97. Epub 2018/02/11. doi: 10.1002/mrm.27121. PubMed PMID: 29427295; PubMed Central PMCID: PMCPMC6085175.

21. Nagel AM, Lehmann-Horn F, Weber MA, Jurkat-Rott K, Wolf MB, Radbruch A, et al. In vivo 35Cl MR imaging in humans: a feasibility study. *Radiology* (2014) 271(2):585-95. Epub 2014/02/06. doi: 10.1148/radiol.13131725. PubMed PMID: 24495267.

22. Biller A, Badde S, Nagel A, Neumann JO, Wick W, Hertenstein A, et al. Improved Brain Tumor Classification by Sodium MR Imaging: Prediction of IDH Mutation Status and Tumor Progression. *AJNR Am J Neuroradiol* (2016) 37(1):66-73. Epub 2015/10/24. doi: 10.3174/ajnr.A4493. PubMed PMID: 26494691; PubMed Central PMCID: PMCPMC7960203.

23. Regnery S, Behl NGR, Platt T, Weinfurtner N, Windisch P, Deike-Hofmann K, et al. Ultra-high-field sodium MRI as biomarker for tumor extent, grade and IDH mutation status in glioma patients. *Neuroimage Clin* (2020) 28:102427. Epub 2020/10/02. doi: 10.1016/j.nicl.2020.102427. PubMed PMID: 33002860; PubMed Central PMCID: PMCPMC7527584.

24. Paech D, Regnery S, Platt T, Behl NGR, Weckesser N, Windisch P, et al. Assessment of Sodium MRI at 7 Tesla as Predictor of Therapy Response and Survival in Glioblastoma Patients. *Front Neurosci* (2021) 15:782516. Epub 2021/12/21. doi: 10.3389/fnins.2021.782516. PubMed PMID: 34924945; PubMed Central PMCID: PMCPMC8671745.

25. Hoffmann SH, Begovatz P, Nagel AM, Umathum R, Schommer K, Bachert P, et al. A measurement setup for direct 17O MRI at 7 T. *Magn Reson Med* (2011) 66(4):1109-15. Epub 2011/03/12. doi: 10.1002/mrm.22871. PubMed PMID: 21394777.

26. Paech D, Nagel AM, Schultheiss MN, Umathum R, Regnery S, Scherer M, et al. Quantitative Dynamic Oxygen 17 MRI at 7.0 T for the Cerebral Oxygen Metabolism in Glioma. *Radiology* (2020) 295(1):181-9. Epub 2020/02/19. doi: 10.1148/radiol.2020191711. PubMed PMID: 32068505.

27. Meissner JE, Korzowski A, Regnery S, Goerke S, Breitling J, Floca RO, et al. Early response assessment of glioma patients to definitive chemoradiotherapy using chemical exchange saturation transfer imaging at 7 T. *J Magn Reson Imaging* (2019) 50(4):1268-77. Epub 2019/03/14. doi: 10.1002/jmri.26702. PubMed PMID: 30864193.

28. Zaiss M, Windschuh J, Paech D, Meissner JE, Burth S, Schmitt B, et al. Relaxation-compensated CEST-MRI of the human brain at 7T: Unbiased insight into NOE and amide signal changes in human glioblastoma. *Neuroimage* (2015) 112:180-8. Epub 2015/03/03. doi: 10.1016/j.neuroimage.2015.02.040. PubMed PMID: 25727379.

29. Dreher C, Oberhollenzer J, Meissner JE, Windschuh J, Schuenke P, Regnery S, et al. Chemical exchange saturation transfer (CEST) signal intensity at 7T MRI of WHO IV degrees gliomas is dependent on the anatomic location. *J Magn Reson Imaging* (2019) 49(3):777-85. Epub 2018/08/23. doi: 10.1002/jmri.26215. PubMed PMID: 30133046.

30. Paech D, Windschuh J, Oberhollenzer J, Dreher C, Sahm F, Meissner JE, et al. Assessing the predictability of IDH mutation and MGMT methylation status in glioma patients using relaxation-compensated multipool CEST MRI at 7.0 T. *Neuro Oncol* (2018) 20(12):1661-71. Epub 2018/05/08. doi: 10.1093/neuonc/noy073. PubMed PMID: 29733378; PubMed Central PMCID: PMCPMC6231210.

31. Heo HY, Jones CK, Hua J, Yadav N, Agarwal S, Zhou J, et al. Whole-brain amide proton transfer (APT) and nuclear overhauser enhancement (NOE) imaging in glioma patients using low-power steady-state pulsed chemical exchange saturation transfer (CEST) imaging at 7T. *J Magn Reson Imaging* (2016) 44(1):41-50. Epub 2015/12/15. doi: 10.1002/jmri.25108. PubMed PMID: 26663561; PubMed Central PMCID: PMCPMC4902781.

32. Windschuh J, Zaiss M, Meissner JE, Paech D, Radbruch A, Ladd ME, et al. Correction of B1-inhomogeneities for relaxation-compensated CEST imaging at 7 T. *NMR Biomed* (2015) 28(5):529-37. Epub 2015/03/20. doi: 10.1002/nbm.3283. PubMed PMID: 25788155.

33. Zaiss M, Windschuh J, Goerke S, Paech D, Meissner JE, Burth S, et al. Downfield-NOE-suppressed amide-CEST-MRI at 7 Tesla provides a unique contrast in human glioblastoma. *Magn Reson Med* (2017) 77(1):196-208. Epub 2016/02/05. doi: 10.1002/mrm.26100. PubMed PMID: 26845067.

34. Liu D, Zhou J, Xue R, Zuo Z, An J, Wang DJ. Quantitative characterization of nuclear overhauser enhancement and amide proton transfer effects in the human brain at 7 tesla. *Magn Reson Med* (2013) 70(4):1070-81. Epub 2012/12/15. doi: 10.1002/mrm.24560. PubMed PMID: 23238951; PubMed Central PMCID: PMCPMC3605209.

35. Paech D, Zaiss M, Meissner JE, Windschuh J, Wiestler B, Bachert P, et al. Nuclear overhauser enhancement mediated chemical exchange saturation transfer imaging at 7 Tesla in glioblastoma patients. *PLoS One* (2014) 9(8):e104181. Epub 2014/08/12. doi: 10.1371/journal.pone.0104181. PubMed PMID: 25111650; PubMed Central PMCID: PMCPMC4128651.

36. Paech D, Burth S, Windschuh J, Meissner JE, Zaiss M, Eidel O, et al. Nuclear Overhauser Enhancement imaging of glioblastoma at 7 Tesla: region specific correlation with apparent diffusion coefficient and histology. *PLoS One* (2015) 10(3):e0121220. Epub 2015/03/20. doi: 10.1371/journal.pone.0121220. PubMed PMID: 25789657; PubMed Central PMCID: PMCPMC4366097.

37. Paech D, Dreher C, Regnery S, Meissner JE, Goerke S, Windschuh J, et al. Relaxation-compensated amide proton transfer (APT) MRI signal intensity is associated with survival and progression in high-grade glioma patients. *Eur Radiol* (2019) 29(9):4957-67. Epub 2019/02/28. doi: 10.1007/s00330-019-06066-2. PubMed PMID: 30809720.

38. Neal A, Moffat BA, Stein JM, Nanga RPR, Desmond P, Shinohara RT, et al. Glutamate weighted imaging contrast in gliomas with 7Tesla magnetic resonance imaging. *Neuroimage Clin* (2019) 22:101694. Epub 2019/03/02. doi: 10.1016/j.nicl.2019.101694. PubMed PMID: 30822716; PubMed Central PMCID: PMCPMC6396013.

39. Xu X, Yadav NN, Knutsson L, Hua J, Kalyani R, Hall E, et al. Dynamic Glucose-Enhanced (DGE) MRI: Translation to Human Scanning and First Results in Glioma Patients. *Tomography* (2015) 1(2):105-14. Epub 2016/01/19. doi: 10.18383/j.tom.2015.00175. PubMed PMID: 26779568; PubMed Central PMCID: PMCPMC4710854.

40. Paech D, Schuenke P, Koehler C, Windschuh J, Mundiyanapurath S, Bickelhaupt S, et al. T1rho-weighted Dynamic Glucose-enhanced MR Imaging in the Human Brain. *Radiology* (2017) 285(3):914-22. Epub 2017/06/20. doi: 10.1148/radiol.2017162351. PubMed PMID: 28628422.

41. Schuenke P, Koehler C, Korzowski A, Windschuh J, Bachert P, Ladd ME, et al. Adiabatically prepared spin-lock approach for T1rho-based dynamic glucose enhanced MRI at ultrahigh fields. *Magn Reson Med* (2017) 78(1):215-25. Epub 2016/08/16. doi: 10.1002/mrm.26370. PubMed PMID: 27521026.

42. Schuenke P, Paech D, Koehler C, Windschuh J, Bachert P, Ladd ME, et al. Fast and Quantitative T1rho-weighted Dynamic Glucose Enhanced MRI. *Sci Rep* (2017) 7:42093. Epub 2017/02/09. doi: 10.1038/srep42093. PubMed PMID: 28169369; PubMed Central PMCID: PMCPMC5294399.

43. Radbruch A, Eidel O, Wiestler B, Paech D, Burth S, Kickingereder P, et al. Quantification of tumor vessels in glioblastoma patients using time-of-flight angiography at 7 Tesla: a feasibility study. *PLoS One* (2014) 9(11):e110727. Epub 2014/11/22. doi: 10.1371/journal.pone.0110727. PubMed PMID: 25415327; PubMed Central PMCID: PMCPMC4240575.
